# Effects of escitalopram and paroxetine on mTORC1 signaling in the rat hippocampus under chronic restraint stress

**DOI:** 10.1186/s12868-017-0357-0

**Published:** 2017-04-26

**Authors:** Mi Kyoung Seo, Cheol Min Choi, Roger S. McIntyre, Hye Yeon Cho, Chan Hong Lee, Rodrigo B. Mansur, Yena Lee, Jae-Hon Lee, Young Hoon Kim, Sung Woo Park, Jung Goo Lee

**Affiliations:** 1 Paik Institute for Clinical Research, Inje University, 633-165 Gaegum-dong, Jin-gu, Busan, 614-735 Republic of Korea; 2 Department of Health Science and Technology, Graduate School, Inje University, Busan, Republic of Korea; 3 Mood Disorders Psychopharmacology Unit, University Health Network, Toronto, ON Canada; 4 Department of Psychiatry, University of Toronto, Toronto, ON Canada; 5 Department of Psychiatry, Korea University Ansan Hospital, Korea University College of Medicine, Ansan, Republic of Korea; 6 Department of Psychiatry, Gongju National Hospital, Gongju, Republic of Korea; 7 Department of Psychiatry, School of Medicine, Haeundae Paik Hospital, Inje University, Busan, Republic of Korea

**Keywords:** Chronic restraint stress, Hippocampus, mTOR signaling, Antidepressants, Neuroplasticity

## Abstract

**Background:**

Recent studies have suggested that the activation of mammalian target of rapamycin (mTOR) signaling may be related to antidepressant action. Therefore, the present study evaluated whether antidepressant drugs would exert differential effects on mTOR signaling in the rat hippocampus under conditions of chronic restraint stress. Male Sprague–Dawley rats were subjected to restraint stress for 6 h/days for 21 days with either escitalopram (10 mg/kg) or paroxetine (10 mg/kg) administered after the chronic stress procedure. Western blot analyses were used to assess changes in the levels of phospho-Ser^2448^-mTOR, phospho-Thr^37/46^-4E-BP-1, phospho-Thr^389^-p70S6 K, phospho-Ser^422^-eIF4B, phospho-Ser^240/244^-S6, phospho-Ser^473^-Akt, and phospho-Thr^202^/Tyr^204^-ERK in the hippocampus.

**Results:**

Chronic restraint stress significantly decreased the levels of phospho-mTOR complex 1 (mTORC1), phospho-4E-BP-1, phospho-p70S6 K, phospho-eIF4B, phospho-S6, phospho-Akt, and phospho-ERK (*p* < 0.05); the administration of escitalopram and paroxetine increased the levels of all these proteins (*p* < 0.05 or 0.01). Additionally, chronic restraint stress reduced phospho-mTORC1 signaling activities in general, while escitalopram and paroxetine prevented these changes in phospho-mTORC1 signaling activities.

**Conclusion:**

These findings provide further data that contribute to understanding the possible relationships among mTOR activity, stress, and antidepressant drugs.

**Electronic supplementary material:**

The online version of this article (doi:10.1186/s12868-017-0357-0) contains supplementary material, which is available to authorized users.

## Background

Depression is a chronic mental illness that involves multiple episodes [1]. The lifetime prevalence of depression in the United States has been estimated at up to 17% [2]. Moreover, this disorder is associated with substantial morbidity, reduced quality of life, and premature mortality [3, 4]. As a result, determining the underlying pathoetiological substrates of mood disorders continues to be a focus of ongoing research [5].

Meaningful advances have been made towards identifying the brain regions and neural circuits that may regulate emotions, mood, and anxiety [6]. Additionally, several neurochemical and molecular changes that underlie depression and stress-related disorders have been observed [7]. For example, stress can induce activation of the hypothalamic–pituitary–adrenal (HPA) axis and increase glucocorticoid hormone production during adaptive responses [8]. Thus, the HPA system has a significant impact on the brain and its major functions, such as mood, cognition, and behavior [9]. A remarkable finding regarding the influence of stress on the brain is that the stress response can result in neurodegeneration [10], including decreased brain volume, neuronal atrophy, and decreases in synaptic proteins [11]. Stress is also related to a decrease in the number of spines on neurons [5]. Taken together, these recent findings suggest that stress and depression may cause changes in neuronal and/or glial size, shape, and density in brain regions that regulate mood and emotion [12].

Of the recently reported findings on the molecular mechanisms associated with synaptogenesis, increases in synaptic protein levels after the activation of mammalian target of rapamycin complex 1 (mTORC1) signaling are of particular importance [13]. mTORC1 is a protein serine/threonine kinase that belongs to the phosphatidylinositol 3-kinase (PI3K)-related kinase family and in involved in a variety of biological processes [14]. Two structurally and functionally distinct mTOR-containing complexes have been identified. The defining components of the first, mTORC1, include the regulatory association protein of mTOR (Raptor) and the proline-rich Akt substrate 40 kDa [PRAS40; 15]; the activity of mTORC1 is specifically sensitive to rapamycin. The second complex, mTORC2, is composed of the rapamycin-insensitive companion of mTOR (Rictor), mammalian stress-activated mitogen-activated protein (MAP) kinase-interacting protein 1 (mSin1), and proteins observed with Rictor 1 and 2 (Protor-1 and Protor-2) [14]. mTORC1 is a regulator of cell growth and metabolism, while mTORC2 may be related to cell survival and cytoskeletal organization [16].

Li et al. [13] reported that a sub-anesthetic dose of ketamine (10 mg/kg) in mice increases mTOR phosphorylation and the levels of synaptic proteins, such as postsynaptic density protein 95 (PSD95), glutamate ionotropic receptor *α*‐amino‐3‐hydroxy‐5‐methylisoxazole‐4‐propionic acid (AMPA) type subunit 1 (GluA1), and synapsin I in the prefrontal cortex. These mice also exhibited decreases in immobility time in the forced swimming test (FST) and increases in synaptic protein levels when the ketamine treatment was blocked by rapamycin [13]. Therefore, the increased levels of synaptic proteins after ketamine treatment may be attributable to mTORC1 signaling activation. Park et al. [17] also observed differential influences of antidepressants on mTORC1 phosphorylation, synaptic protein expression, and neurite outgrowth under toxic conditions in rat primary hippocampal neurons.

**Fig. 1:**
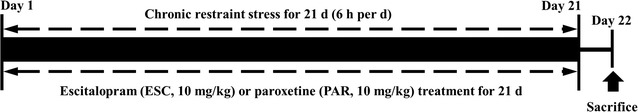
Schematic diagram of the experimental schedule. Escitalopram (ESC, 10 mg/kg) or paroxetine (PAR, 10 mg/kg) was administered 1 h prior to restraint stress for a total of 21 days (6 h/days). The rats were sacrificed on the 22nd day

The present study sought to assess whether antidepressants would exert varying effects on mTOR signaling in the rat hippocampus under conditions of chronic stress. A 21-day chronic restraint model was employed as the stress condition and the phosphorylation levels of mTORC1 upstream regulators (Akt and extracellular signal regulated protein [ERK]) and downstream effectors (eukaryotic translation initiation factor 4E binding protein 1 [4E-BP-1], p70 ribosomal S6 kinase [p70S6 K], eukaryotic translation initiation factor 4B [eIF4B], and small ribosomal protein 6 [S6]) in the rat hippocampus were assessed with Western blot analyses (Fig. 1).

## Results

### Effects of antidepressants on the expression of phosphorylated mTORC1 following chronic restraint stress

**Fig. 2:**
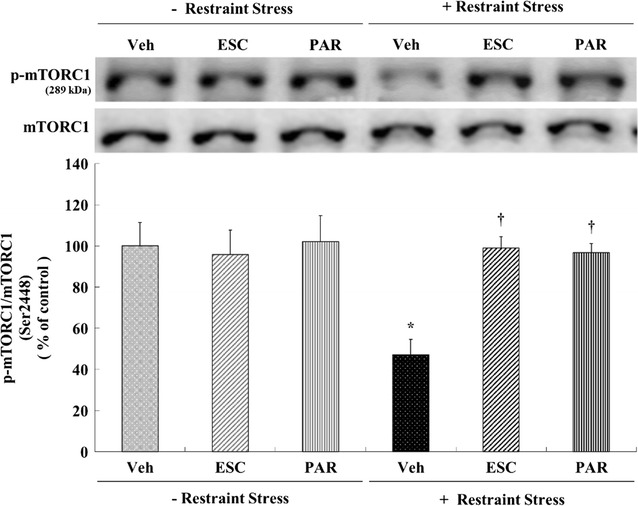
Effects of antidepressants on levels of phospho-mTORC1 in the rat hippocampus. Rats (*n* = 6 animals/group) were given a daily injection of vehicle (Veh; 1 mL/kg), ESC (10 mg/kg), or PAR (10 mg/kg) for 21 days with or without restraint stress (6 h daily for 21 days). Levels of phosphorylated mTORC1 in brain homogenates from the hippocampus were detected by SDS-PAGE and Western blot analyses using anti-phospho-Ser^2448^-mTORC1 antibodies. A representative image and quantitative analysis normalized to the levels of total mTORC1 are shown. Results are expressed as a percentage of vehicle control and represent the mean ± standard error of the mean (SEM) of 6 animals per group. *p < 0.05 versus vehicle control; ^†^ *p* < 0.05 versus stress + vehicle

A two-way analysis of variance (ANOVA; Table 1) was performed to evaluate changes in phosphorylated mTORC1 levels and revealed significant individual effects of stress and drug (escitalopram and paroxetine) as well as significant interactions between stress and drug (stress × escitalopram and stress × paroxetine). Chronic restraint stress significantly decreased phospho-Ser^2448^-mTORC1 expression by 52.78% in the hippocampus compared with the vehicle control group (*p* = 0.013; Fig. 2). Escitalopram and paroxetine markedly prevented the chronic restraint stress-induced decrease in phospho-Ser^2448^-mTORC1 (stress + escitalopram = 98.91% of control, *p* = 0.016; stress + paroxetine = 96.73% of control, *p* = 0.023; Fig. 2). Neither antidepressant affected phospho-Ser^2448^-mTORC1 levels under normal conditions.

**Table 1:** Summary of the two-way analysis of variance on changes in phosphorylated mTORC1, downstream effectors of mTORC1, and upstream activators of mTORC1 related to treatment, stress and on the interaction of treatment and stress

	Escitalopram		Paroxetine	
	*F*	*P*	*F*	*P*
mTORC1				
Drug	7.498	0.013	8.198	0.010
Stress	8.402	0.009	10.616	0.004
Drug × stress	10.662	0.004	6.990	0.016
Downstream effectors of mTORC1				
4E-BP-1				
Drug	13.592	0.001	12.403	0.002
Stress	20.717	<0.001	28.600	<0.001
Drug × stress	9.579	0.006	11.679	0.003
p70S6 K				
Drug	8.868	0.007	5.866	0.025
Stress	17.816	<0.001	17.947	<0.001
Drug × stress	10.815	0.004	7.900	0.011
eIF4B				
Drug	8.157	0.010	6.669	0.018
Stress	19.425	<0.001	16.753	0.001
Drug × stress	5.919	0.024	8.980	0.007
S6				
Drug	4.194	0.050	6.878	0.016
Stress	12.204	0.002	12.117	0.002
Drug × stress	7.238	0.014	14.918	0.001
Upstream activators of mTORC1				
Akt				
Drug	21.744	<0.001	18.971	<0.001
Stress	47.192	<0.001	51.327	<0.001
Drug × stress	17.682	<0.001	13.422	0.002
ERK				
Drug	17.581	<0.001	21.123	<0.001
Stress	14.075	0.001	25.637	<0.001
Drug × stress	21.477	<0.001	14.569	0.001

*mTORC1* mammalian target of rapamycin complex 1, *4E*-*BP*-*1* eukaryotic initiation factor 4E-binding protein 1, *p70S6* *K* p70 ribosomal protein S6 kinase, *eI4FB* eukaryotic translation initiation factor 4B, *S6* small ribosomal protein 6, *ERK* extracellular signal-regulated kinase

### Effects of antidepressants on the expression of phosphorylated mTORC1 downstream effectors (4E-BP-1, p70S6 K, eIF4B, and S6)

**Fig. 3:**
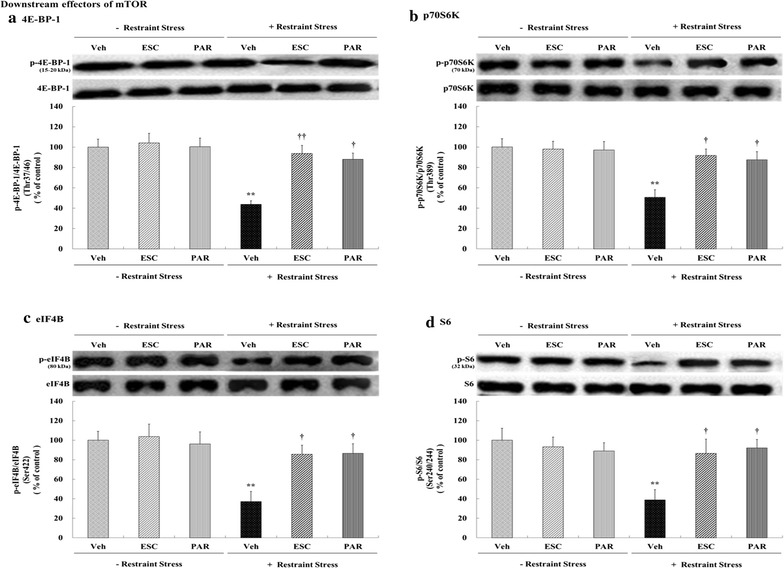
Effects of antidepressants on the levels of mTORC1 downstream effectors (phospho-4E-BP-1, phospho-p70S6 K, phosphor-eIF4B, and phospho-S6) in the rat hippocampus. Rats (*n* = 6 animals/group) were given a daily injection of Veh (1 mL/kg), ESC (10 mg/kg), or PAR (10 mg/kg) for 21 days with or without restraint stress (6 h daily for 21 days). Levels of phosphorylated 4E-BP-1, p70S6 K, eIF4B, and S6 in brain homogenates from the hippocampus were detected by SDS-PAGE and Western blot analyses using anti-phospho-Thr^37/46^-4E-BP-1 (**a**), anti-phospho-Thr^389^-p70S6 K (**b**), anti-phospho-Ser^422^-eIF4B (**c**), and anti-phospho-Ser^240/244^-S6 (**d**) antibodies. A representative image and quantitative analysis normalized to the levels of total 4E-BP-1 (**a**), p70S6 K (**b**), eIF4B (**c**), and S6 (**d**) are shown. The results are expressed as a percentage of vehicle control and represent the mean ± SEM of 6 animals per group. **p < 0.01 versus vehicle control; ^†^ *p* < 0.05 or ^††^ *p* < 0.01 versus stress + vehicle

There were significant individual effects of stress and drug (escitalopram and paroxetine) on the phosphorylated levels of 4E-BP-1, p70S6 K, eIF4B, and S6 (Table 1) as well as an interaction between these two factors (stress × escitalopram and stress × paroxetine) that significantly affected these levels (Table 1). Specifically, chronic restraint stress decreased the expression of mTORC1 downstream regulators and produced significant decreases in the levels of phospho-Thr^37/46^-4E-BP-1 (56.25%, *p* = 0.001, Fig. 3a), phospho-Thr^389^-p70S6 K (49.29%, *p* = 0.002, Fig. 3b), phospho-Ser^422^-eIF4B (62.88%, *p* = 0.005, Fig. 3c), and phospho-Ser^240/244^-S6 (61.24%, *p* = 0.004, Fig. 3d) compared with the vehicle control group. However, treatment with escitalopram and paroxetine prevented the chronic restraint stress-induced decreases in the phosphorylated levels of these mTORC1 downstream effectors (4E-BP-1: stress + escitalopram = 93.69% of control, *p* = 0.003; stress + paroxetine = 88.02% of control, *p* = 0.010, Fig. 3a; p70S6 K: stress + escitalopram = 91.68% of control, *p* = 0.012; stress + paroxetine = 87.40% of control, *p* = 0.034, Fig. 3b; eIF4B: stress + escitalopram = 85.55% of control, *p* = 0.048; stress + paroxetine = 86.56% of control, *p* = 0.042, Fig. 3c; S6: stress + escitalopram = 86.53% of control, *p* = 0.046; stress + paroxetine = 92.09% of control, *p* = 0.014, Fig. 3d). The phosphorylated levels of these mTORC1 downstream effectors were not affected by antidepressants under normal conditions.

### Effects of antidepressants on the expression of phosphorylated mTORC1 upstream activators (Akt and ERK)

**Fig. 4:**
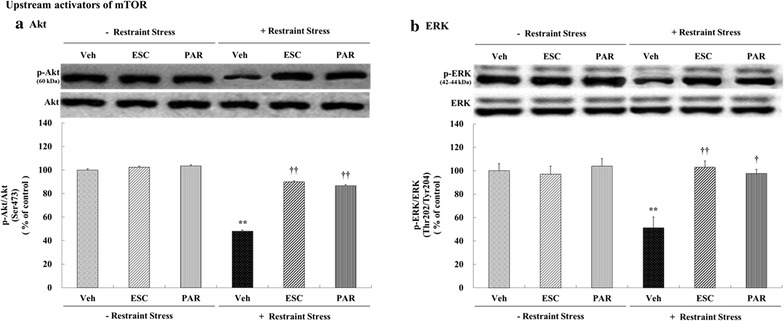
Effects of antidepressants on the levels of mTORC1 upstream activators (phospho-Akt and phospho-ERK) in the rat hippocampus. Rats (*n* = 6 animals/group) were given a daily injection of Veh (1 mL/kg), ESC (10 mg/kg), or PAR (10 mg/kg) for 21 days with or without restraint stress (6 h daily for 21 days). Levels of phosphorylated Akt and ERK in brain homogenates from the hippocampus were detected by SDS-PAGE and Western blot analyses using anti-phospho-Ser^473^-Akt (**a**) and anti-phospho-Thr^202^/Tyr^204^-ERK (**b**) antibodies. A representative image and quantitative analysis normalized to the levels of total Akt (**a**) and ERK (**b**) are shown. The results are expressed as the percentage of vehicle control and represent the mean ± SEM of 6 animals per group. **p < 0.01 versus vehicle controls; ^†^ *p* < 0.05 or ^††^ *p* < 0.01 versus stress + vehicle

Stress, drug (i.e., escitalopram and paroxetine), and their interaction (stress × escitalopram and stress × paroxetine) had significant effects on the levels of phosphorylated Akt and ERK (Table 1). Specifically, chronic restraint stress decreased mTORC1 upstream activators and significantly decreased the levels of phospho-Ser^473^-Akt (47.88% of control, *p* < 0.001, Fig. 4a) and phospho-^Thr202/Tyr204^-ERK (51.22% of control, *p* < 0.001, Fig. 4b) in the hippocampus. However, treatment with escitalopram and paroxetine prevented the chronic restraint stress-induced decreases in the phosphorylated levels of mTORC1 upstream activators (Akt: stress + escitalopram = 89.92% of control, *p* < 0.001; stress + paroxetine = 86.54% of control, *p* = 0.001, Fig. 4a; ERK: stress + escitalopram = 102.91% of control, *p* < 0.001; stress + paroxetine = 97.57% of control, *p* < 0.001, Fig. 4b). These levels were not affected under normal conditions.

## Discussion

**Fig. 5:**
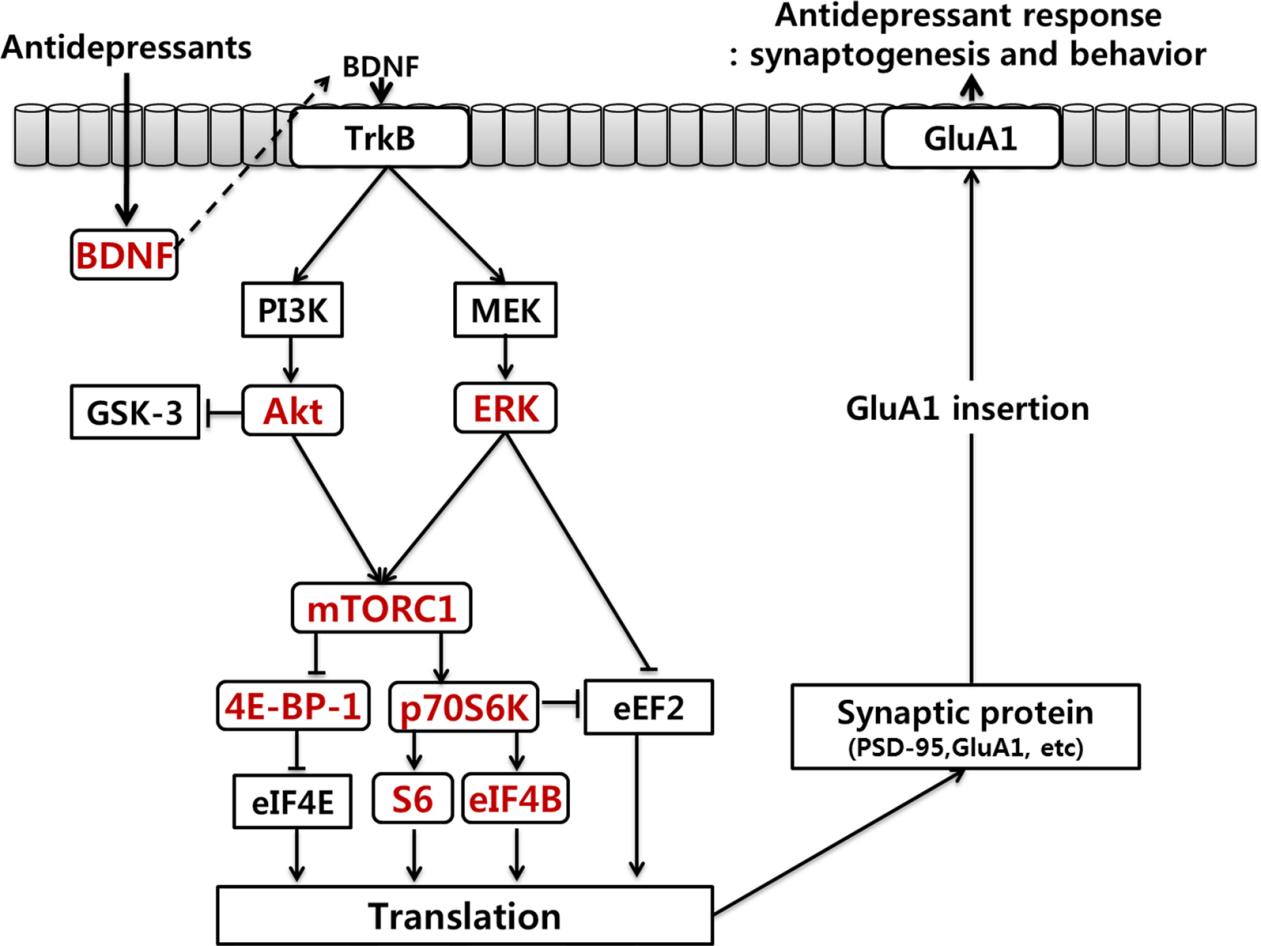
Possible mechanisms underlying antidepressant-induced molecular changes related to antidepressant effects. Antidepressants increase BDNF [please spell out] expression. The release of BDNF and the stimulation of associated signaling cascades (PI3 K/Akt and MEK/ERK) activate mTORC1 signaling and translation which, in turn, increases synaptic protein levels and synaptogenesis. These effects contribute to the sustained antidepressant actions of antidepressants. *TrkB* tyrosine-related kinase B, *PI3* *K* phosphoinositide 3-kinase, *MEK* MAP/ERK kinase, *ERK* extracellular signal-regulated kinases, *GSK*-*3*, glycogen synthase kinase-3, *mTORC1* mammalian target of rapamycin complex 1, *4E*-*BP*-*1* 4E-binding protein 1, p70S6 K p70ribosomal protein S6 kinase, *eEF2* eukaryotic elongation factor 2, *eIF4E* eukaryotic translation initiation factor 4E, *S6* small ribosomal protein 6, *eIF4B* eukaryotic translation initiation factor 4B, *PSD*-*95* post-synaptic density 95, *GluA1* glutamate ionotropic receptor AMPA type subunit 1, *BDNF* brain-derived neurotrophic factor. The molecular pathways shown in *red* illustrate novel observations from the present study while those in *black* are generally accepted signaling pathways involved in antidepressant action

The main findings of this study were that chronic restraint stress decreased the expression of phospho-mTORC1, phospho-4E-BP-1, phospho-p70S6 K, phospho-eIF4B, phospho-S6, phospho-Akt, and phospho-ERK in the rat hippocampus. Additionally, this study showed that escitalopram and paroxetine prevented changes in the levels of phospho-mTORC1, phospho-4E-BP1, phospho-p70S6 K, phospho-eIF4B, phospho-S6, phospho-Akt, and phospho-ERK that were induced by chronic restraint stress. Therefore, escitalopram and paroxetine activated mTORC1 signaling pathways in the rat hippocampus under chronic restraint conditions (Fig. 5).

Stress can facilitate the activity of the HPA axis and the production of glucocorticoids, which are the major stress-reactive hormones [8]. Heightened levels of glucocorticoid hormones may cause neuronal toxicity in certain brain structures and have been associated with mood and emotional dysregulation [18]. However, the underlying cellular mechanisms mediated by stress are not fully understood [19].

Stress can also reduce the expression of growth factors, such as brain-derived neurotrophic factor (BDNF), which may affect neurogenesis in the brain, especially the hippocampus [5, 11, 20]. The hippocampus is a limbic structure implicated in the pathogenesis of mood disorders and related symptoms [9, 11, 20] that establishes circuits with other brain structures, such as the amygdala and prefrontal cortex, and affects learning, memory, and regulation of the HPA axis [18, 21]. The hippocampus also contains considerable quantities of glucocorticoid receptors [22, 23]. Thus, stress can induce neuronal damage and atrophy in the hippocampus as well as cause changes in its structure [24–26].

Magnetic resonance imaging studies have shown that reductions in the hippocampal volume of patients with depression are associated with more frequent episodes [27] and a meta-analysis observed reduced hippocampal volume in patients with unipolar depression [28]. A loss of hippocampal volume has also been observed in patients with first-episode depression [29] and it has been suggested that reduced hippocampal volume might be a biomarker of the progression of depression [29, 30]. Taken together, these findings suggest that the pathophysiology of depression may be associated with the decreased volumes of cortical and limbic brain regions, atrophy of neurons, and decreased numbers of synaptic connections [25, 31, 32].

As mentioned above, stress reduces the expression and function of BDNF in brain structures related to the pathogenesis of depression. Reduced levels of BDNF or growth factors may be related to the structural and neural plastic changes associated with stress and depression [32, 33] because decreases in BDNF may cause neuronal death and atrophy; this factor is necessary for neuronal remodeling. An increased vulnerability to depression-like behaviors was observed in BDNF-heterozygous knockout mice [34, 35], while human studies have reported that the presence of the BDNF Val66Met allele blocks the normal maturation of BDNF and may cause neuronal atrophy in hippocampal neurons [36].

These effects may be due to the modification of intracellular signaling pathways by BDNF. The major intracellular signaling pathways involved in neuronal survival and synaptogenesis are the PI3 K-Akt and mitogen-activated protein kinase (MAPK) signaling pathways [37, 38], which have multiple downstream targets that regulate neuronal survival, neuroprotection, and synaptic plasticity [39, 40]. An important downstream target for the regulation of synaptic plasticity and production of synaptic proteins is mTORC1 [13, 14, 32]. Neurotrophic factors regulate mTORC1 signaling; however, one’s nutritional, energy, endocrine, and metabolic status can also regulate mTORC1 signaling activity [40, 42]. For example, the expression of mTORC1 in primary rat hippocampal neurons decreases under B27-deprivation conditions [17], while treadmill exercise increases the level of mTORC1 and synaptic proteins in the rat hippocampus following 7 days of immobilization stress [41]. Additionally, ketamine increases mTORC1 activity and the production of synaptic proteins in the mouse prefrontal cortex and rat primary hippocampal neurons [5, 13, 17, 32]. Therefore, it is possible that mTORC1 is a convergence pathway for synaptic plasticity and the production of synaptic proteins [5, 32, 43].

Chronic restraint stress is one experimental method that can be used to create stressful conditions in animals [44]. Therefore, the present study adopted a repeated restraint stress paradigm [6 h/days for 21 days; 45, 46]. Previous studies have shown that chronic restraint stress decreased the levels of BDNF, PSD95, and β-catenin in the rat hippocampus [47] and resulted in the retraction of dendrites in hippocampal CA3 neurons and spatial memory deficits in rats [48]. A murine study reported that chronic restraint stress impaired neurogenesis in the hippocampus and produced hippocampus-dependent fear memory [19]. Similarly, the use of a 7-day immobilization stress paradigm decreased levels of synaptic proteins, such as PSD95 and synaptophysin [41].

In a previous study, 8 weeks of chronic unpredictable stress resulted in reduced levels of phosphorylated mTORC1, ERK-1/2, Akt1, and GluA1 in the rat amygdala [49]. However, there were no significant changes in these proteins in the frontal cortex, hippocampus, or dorsal raphe [49]. These discrepant results may be due to the different types of stressors and varying periods of stress used in the experiments. Similarly, 21 days of chronic unpredictable stress decreased the expression levels of PSD95, GluA1, and synapsin I, as well as decreased the number of spines and inhibited excitatory postsynaptic currents in the rat prefrontal cortex [50]. Moreover, 21 days of chronic unpredictable stress decreased mTORC1 expression and increased levels of regulated in development and DNA damage response-1 (REDD1), which is an inhibitor of mTORC1 in the rat prefrontal cortex [51]. Although a different stress paradigm was used in the present study, decreased levels of phospho-mTORC1 and its downstream effectors phospho-4E-BP-1, phospho-p70S6 K, phospho-eIF4B, and phospho-S6 were observed in the rat hippocampus. Furthermore, there were decreased expression levels of phospho-Akt and phospho-ERK, which are upstream activators of mTORC1.

Activated mTORC1 phosphorylates 4E-BP and p70S6 K [52, 53] and activated p70S6 K phosphorylates S6 and eIF4B [52–54], which subsequently facilitate protein translation [50–52]. Thus, decreased expression levels of mTORC1 may be related to decreased levels of 4E-BP-1, P70S6 K, eIF4B, and S6. Previous studies have reported that 21 days of restraint stress in mice decreased levels of Akt and ERK in the hippocampus and that 7 days of immobilization stress reduced Akt in the rat hippocampus [41, 55]. The decreased levels of Akt and ERK may be related to the lower levels of mTORC1 and the decreased effect on mTORC1 downstream effectors. In the present study, the effects of 21-day chronic restraint stress on the expression of synaptic proteins was not assessed; however, the present and previous studies have shown that 21 days of restraint stress significantly reduces the levels of BDNF, PSD95, and synaptophysin in the rat hippocampus [47, 56]. Therefore, it is possible that chronic restraint stress decreased activation of the mTORC1 signaling pathway in the present study.

The present study also showed that treatment with escitalopram and paroxetine prevented the chronic restraint stress-induced reduction of phospho-mTORC1 expression in the rat hippocampus. Escitalopram and paroxetine also prevented the chronic stress-induced reduction in the levels phospho-4E-BP1, phospho-p70S6 K, phospho-eIF4B, phospho-S6, phospho-Akt, and phospho-ERK. A previous study of rat primary hippocampal neurons showed that escitalopram and paroxetine increased the levels of phospho-mTORC1, phospho-4E-BP-1, phospho-p70S6 K, phospho-eIF4B, and phospho-S6 under B27-deprived toxic conditions [17]. Moreover, escitalopram and paroxetine also increased the levels of phospho-Akt and phospho-ERK [17]. Although different doses of antidepressant drugs were used in the present study, the findings were similar to those of the in vitro study [17]. Therefore, escitalopram and paroxetine could prevent decreases in the levels of mTORC1 as well as its downstream effectors and upstream regulators after chronic restraint stress. In other words, chronic restraint stress could decrease the activation of mTORC1 signaling but this may be prevented by some antidepressant treatments.

To our knowledge, this is the first report of the effects of antidepressants on the mTORC1 signaling pathway in the rat hippocampus. Notwithstanding, this study has several limitations. First, although previous studies have shown that chronic restraint stress induces depression-like behavior in behavioral tests [57–59], the present study did not confirm the behavioral effects of this chronic restraint stress paradigm. Second, the levels of synaptic proteins, such as PSD95, GluA1, and synapsin I, were not assessed in the present study. Third, the effects of mTORC1 inhibitors, such as rapamycin, and other signal pathway inhibitors were not evaluated in the present study. Therefore, additional work that addresses these limitations is needed to strengthen the findings of this study.

## Conclusions

In summary, chronic restraint stress reduced mTORC1 signaling activities in the rat hippocampus but these decreases were prevented by treatment with escitalopram and paroxetine. The findings of this study may allow for a better understanding of the possible relationships between mTOR activity and the biology of stress. Furthermore, the present findings highlight that some antidepressants may regulate mTOR signaling activity in chronic stress situations.

## Methods

### Drugs and reagents

Escitalopram oxalate (Pangbourne, UK), and paroxetine HCl (Holzkirchen, DE) were gifts from Sandoz. The antibodies for the Western blot analyses were obtained from the following sources: anti-phospho-mTOR (Ser2448, #2971), anti-mTOR (#2972), anti-phospho-Akt (Ser473, #9271), anti-Akt (#9272), anti-phospho-p44/42 MAPK (ERK1/2) (Thr202/Tyr204, #9101), anti-p44/42 MAPK (ERK1/2, #4695), anti-phospho-4E-BP-1 (Thr37/46, #2855), anti-4E-BP-1 (#9452), anti-phospho-eIF4B (Ser422, #3591), anti-eIF4B (#3592), anti-phospho-S6 (Ser240/244, #2251), anti-S6 (#2217), anti-phospho-p70S6 K (Thr389, #9205), and anti-p70S6 K (#9202) from Cell Signaling Technology (Beverly, MA, USA); anti-BDNF (sc-546) and goat anti-rabbit IgG-horseradish-peroxide conjugates (sc-2004) from Santa Cruz Biotechnology (Santa Cruz, CA, USA); and monoclonal anti-α-tubulin (T9026) and anti-mouse IgG peroxidase conjugates (A4416) from Sigma (St. Louis, MO, USA).

### Animals

Male Sprague–Dawley rats (Orient Bio, GyeongGi-Do, Korea) weighing 200–250 g were housed 2 or 3 per cage with ad libitum food and water in an environment maintained at 21 °C on a 12/12-h light/dark cycle.

After 7 days of acclimatization, the rats were randomly divided into 6 groups of 6 rats each. All drugs were dissolved in vehicle (0.7% glacial acetic acid in 0.9% saline) and intraperitoneally (i.p.) injected into the animals. The first group (vehicle) received vehicle (1 mL/kg, i.p.) without immobilization stress; the second (escitalopram) and third (paroxetine) groups received escitalopram (10 mg/kg, i.p.) and paroxetine (10 mg/kg, i.p.), respectively, without restraint stress; and the sixth group (vehicle + stress) received the vehicle at 10:00. Then, 1 h later, the rats were completely restrained for 6 h (from 11:00 to 17:00) in specially designed plastic restraint tubes (dimensions: 20-cm high, 7 cm in diameter). The rats in the fourth (escitalopram + stress) and fifth (paroxetine + stress) groups received escitalopram (10 mg/kg, i.p.) or paroxetine (10 mg/kg, i.p.), respectively, and were then restrained in the same way as the rats in the sixth group. These procedures were repeated once daily for 3 weeks (Fig. 1).

The dose of escitalopram (10 mg/kg) used in the present study was selected based on a report showing that this dose exerted antidepressant-like effects in rats with depression-like behaviors induced by chronic mild stress [60]. In rats receiving chronic treatment with paroxetine (3 weeks, 10 mg/kg), the hippocampus exhibited increases in BDNF expression and synaptic levels of AMPA receptor subunits [GluA1 and GluA2/3; 61]. In particular, these doses of escitalopram and paroxetine significantly prevented chronic restraint stress-induced decreases in BDNF mRNA in the rat hippocampus (Additional file 1: Figure S1).

### Protein extraction and Western blotting

The rats were sacrificed by rapid decapitation, 24 h after the final restraint session. Immediately after decapitation and rapid removal of the brain, hippocampus was dissected out. The detailed procedure for western blot analysis was described previously [47].

The membranes were probed with antibodies against anti-phospho-mTORC1 (Ser2448), anti-mTORC1, anti-phospho-Akt (Ser473), anti-Akt, anti-phospho-p44/42 MAPK (ERK1/2) (Thyr202/Tyr204), anti-p44/42 MAPK (ERK1/2), anti-phosho-4E-BP-1 (Thr37/46), anti-4E-BP-1, anti-phospho-p70S6 K (Thr389), anti-p70S6 K, anti-phosho-eIF4B (Ser422), anti-eIF4B, anti-phosho-S6 (Ser240/244), anti-S6, anti-BDNF, 1:1000; and anti-α-tubulin, 1:2000. The membranes were subsequently probed with horseradish peroxidase-conjugated secondary antibody, goat-anti-rabbit IgG for anti-phospho-mTORC1 (Ser2448), anti-mTORC1, anti-phospho-Akt (Ser473), anti-Akt, anti-phospho-p44/42 MAPK (ERK1/2) (Thyr202/Tyr204), anti-p44/42 MAPK (ERK1/2), anti-phosho-4E-BP-1 (Thr37/46), anti-4E-BP-1, anti-phospho-p70S6 K (Thr389), anti-p70S6 K, anti-phosho-eIF4B (Ser422), anti-eIF4B, anti-phosho-S6 (Ser240/244), anti-S6, 1:1000; anti-BDNF, 1:2000; and anti-mouse IgG for anti-α-tubulin 1:10,000. Proteins were detected by Pico EPC Western blot reagents (ELPIS, Daejeon, Korea).

### Statistical analysis

To determine the individual and interactive effects of drug administration and restraint stress on the protein levels, a two-way ANOVA was performed with Scheffe’s tests for post hoc comparisons. A *p* value < 0.05 was considered to indicate statistical significance.

## Additional file

**Additional file 1: Figure S1.** Effects of antidepressants on levels of brain-derived neurotrophic factor (BDNF) in the rat hippocampus. Rats (*n* = 6 animals/group) were given a daily injection of vehicle (Veh; 1 mL/kg), escitalopram (ESC; 10 mg/kg, **a**), or paroxetine (PAR; 10 mg/kg, **b**) for 21 days with or without restraint stress (6 h daily for 21 days). Levels of BDNF in brain homogenates from the hippocampus were detected by SDS-PAGE and Western blot analyses using a BDNF antibody. A representative image and quantitative analysis normalized to α-tubulin are shown. The results are expressed as a percentage of vehicle control and represent the mean ± SEM of 6 animals per group. **p* < 0.05 or ***p* < 0.01 versus vehicle control; ^††^ *p* < 0.01 versus stress + vehicle.

## Abbreviations

4E-BP-1: eukaryotic translation initiation factor 4E binding protein 1
ANOVA: analysis of variance
BNDF: brian-derived neurotrophic factor
eIF4B: eukaryotic translation initiation factor 4B
ERK: extracellular signal regulated protein
FST: forced swimming test
GluR1: glutamate ionotropic receptor α‐amino‐3‐hydroxy‐5‐methylisoxazole‐4‐propionic acid (AMPA) type subunit 1
HPA: hypothalamic-pituitary-adrenal
MAP: mitogen-activated protein
MAPK: mitogen-activated protein kinase
mSin1: mitogen-activated protein kinase-interacting protein 1
mTOR: mammalian target of rapamycin
mTORC1: mammalian target of rapamycin complex 1
mTOR2: mammalian target of rapamycin complex 2
p70S6K: p70 ribosomal S6 kinase
PI3 K: phosphatidylinositol 3-kinase
PRAS40: proline-rich Akt substrate 40 kDa
protor-1: protein obsereved with Rictor 1
protor-2: protein obsereved with Rictor 2
PSD-95: postsynaptic density protein 95
Rator: regulatory association protein of mTOR
REDD1: regulated in development and DNA damage response-1
Rictor: rapamycin-insensitive companion of mTOR
S6: small ribosomal protein 6
