# Supplementary figures and images for: Effects of escitalopram and paroxetine on mTORC1 signaling in the rat hippocampus under chronic restraint stress
Source: BMC Neurosci. 2017 Apr 26;18:39. doi: 10.1186/s12868-017-0357-0 (PMC5405541; doi:10.1186/s12868-017-0357-0)

**Supplementary Figures.**

**
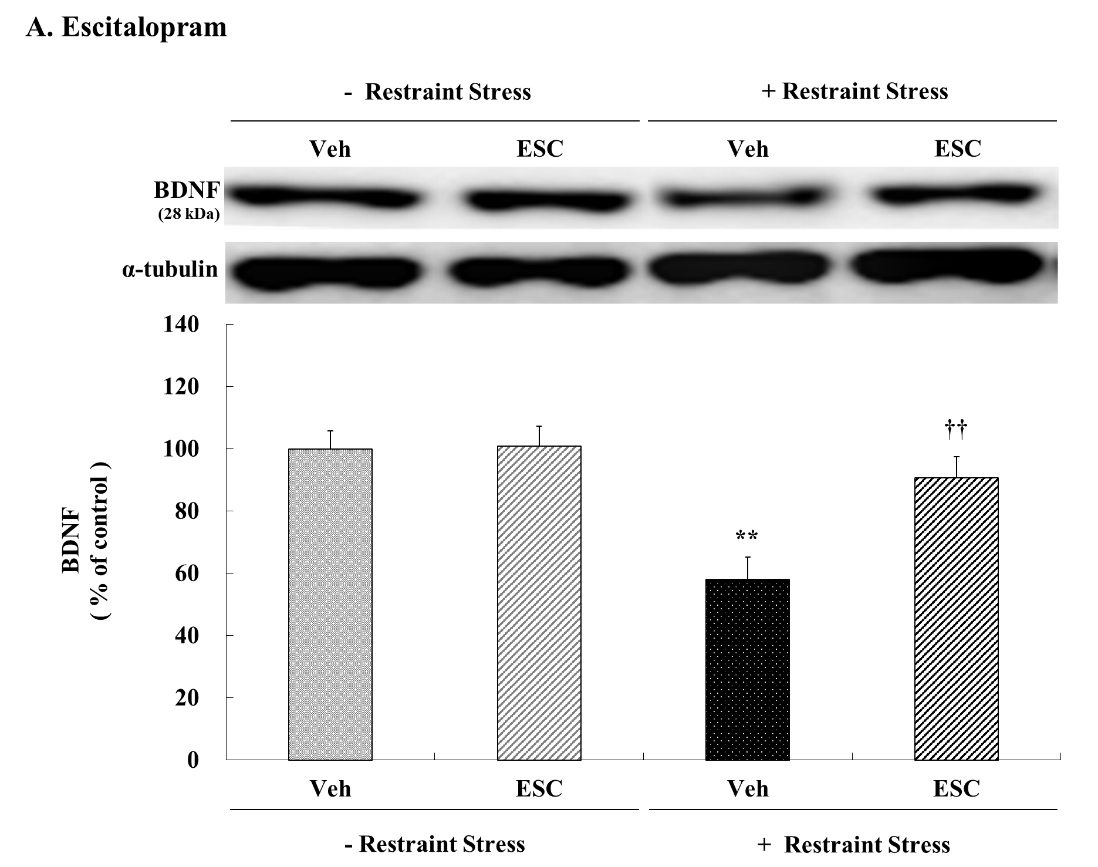
**

**
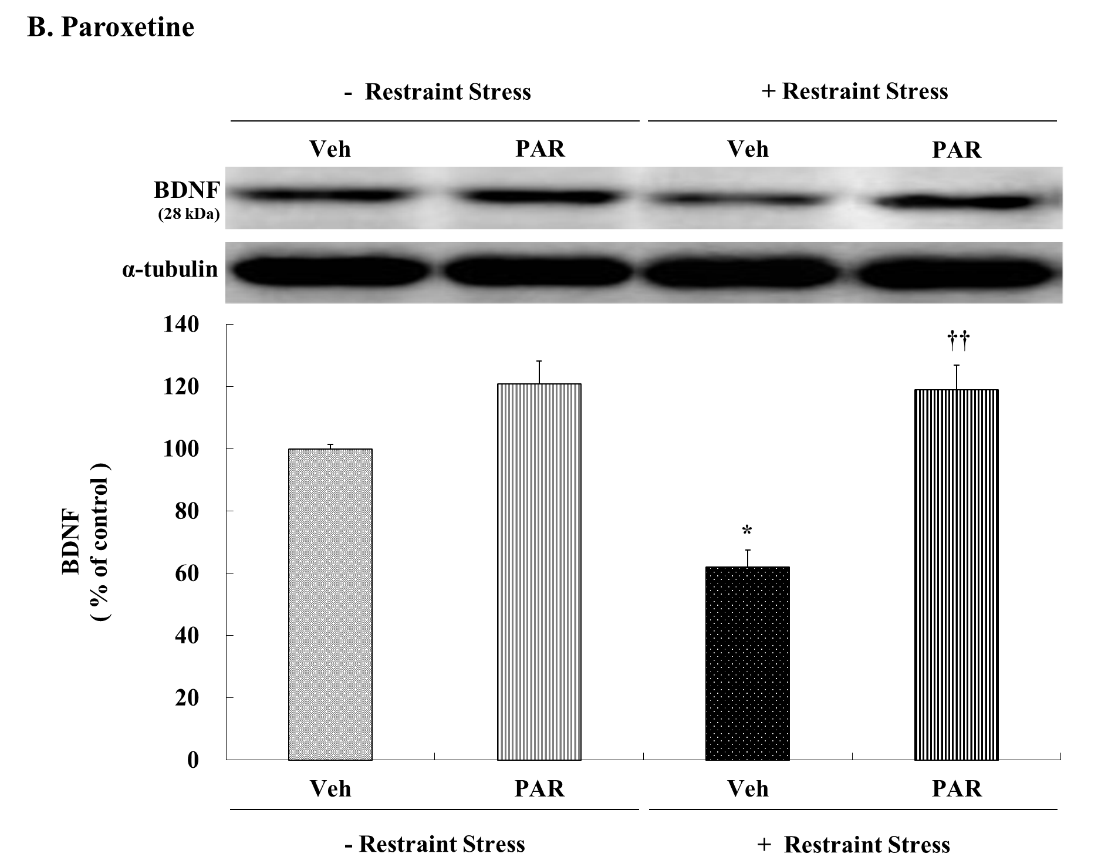
**

Supplement: Supplementary file 1 — Additional file 1: Figure S1. Effects of antidepressants on levels of brain-derived neurotrophic factor (BDNF) in the rat hippocampus. Rats (n = 6 animals/group) were given a daily injection of vehicle (Veh; 1 mL/kg), escitalopram (ESC; 10 mg/kg, a), or paroxetine (PAR; 10 mg/kg, b) for 21 days with or without restraint stress (6 h daily for 21 days). Levels of BDNF in brain homogenates from the hippocampus were detected by SDS-PAGE and Western blot analyses using a BDNF antibody. A representative image and quantitative analysis normalized to α-tubulin are shown. The results are expressed as a percentage of vehicle control and represent the mean ± SEM of 6 animals per group. *p < 0.05 or **p < 0.01 versus vehicle control; †† p < 0.01 versus stress + vehicle. [file 12868_2017_357_MOESM1_ESM.docx]
